# Exposure of wetlands important for nonbreeding waterbirds to sea‐level rise in the Mediterranean

**DOI:** 10.1111/cobi.14288

**Published:** 2024-05-16

**Authors:** Fabien Verniest, Thomas Galewski, Olivier Boutron, Laura Dami, Pierre Defos du Rau, Anis Guelmami, Romain Julliard, Nadège Popoff, Marie Suet, Loïc Willm, Wed Abdou, Hichem Azafzaf, Nadjiba Bendjedda, Taulant Bino, John J. Borg, Luka Božič, Mohamed Dakki, Rhimou El Hamoumi, Vitor Encarnação, Kiraz Erciyas‐Yavuz, Khaled Etayeb, Valeri Georgiev, Ayman Hamada, Ohad Hatzofe, Christina Ieronymidou, Tom Langendoen, Tibor Mikuska, Blas Molina, Filipe Moniz, Caroline Moussy, Asmaâ Ouassou, Nicky Petkov, Danae Portolou, Tareq Qaneer, Samir Sayoud, Marko Šćiban, Goran Topić, Danka Uzunova, Gal Vine, Andrej Vizi, Erald Xeka, Marco Zenatello, Elie Gaget, Isabelle Le Viol

**Affiliations:** ^1^ Tour du Valat Institut de recherche pour la conservation des zones humides méditerranéennes Arles France; ^2^ Centre d'Ecologie et des Sciences de la Conservation (CESCO), Muséum national d'Histoire naturelle, Centre National de la Recherche Scientifique Sorbonne Université, Station Marine de Concarneau Concarneau Cedex France; ^3^ Office Français de la Biodiversité Arles France; ^4^ Aquabio, 108 Av. du Lac Léman La Motte‐Servolex France; ^5^ Egyptian Environmental Affairs Agency El Maadi Helwan Egypt; ^6^ Association “Les Amis des Oiseaux” (AAO/BirdLife en Tunisie) Ariana Tunisia; ^7^ Direction générale des Forêts Ben Aknoun Algeria; ^8^ Albanian Ornithological Society, “Ymer Kurti”, Olympia Center Tirana Albania; ^9^ National Museum of Natural History, Vilhena Palace Mdina Malta; ^10^ DOPPS ‐ Birdlife Slovenia Ljubljana Slovenia; ^11^ Groupe de Recherche pour la Protection des Oiseaux au Maroc (GREPOM), Résidence Oum Hani IV Salé Morocco; ^12^ Ecology and Environment Laboratory, Faculty of Sciences Ben M'sik University Hassan II of Casablanca Casablanca Morocco; ^13^ Instituto da Conservação da Natureza e das Florestas, IP (ICNF) Centro de Estudos de Migrações e Proteção de Aves (CEMPA) Lisboa Portugal; ^14^ Ornithological Research Center Ondokuz Mayis University Samsun Turkey; ^15^ Zoology Department Faculty of Science the University of Tripoli. Alfornaj Tripoli Libya; ^16^ Ministry of Environment and Water National Nature Protection Service Directorate Sofia Bulgaria; ^17^ Israel Nature and Parks Authority Headquarters Am V'Olamo 3 Jerusalem Israel; ^18^ BirdLife Cyprus Nicosia Cyprus; ^19^ Wetlands International Ede The Netherlands; ^20^ Croatian Society for Bird and Nature Protection Osijek Croatia; ^21^ Sociedad Española de Ornitología (SEO/BirdLife) Madrid Spain; ^22^ Instituto da Conservação da Natureza e das Florestas Lisbon Portugal; ^23^ LPO‐BirdLife France, Fonderies Royales Rochefort Cedex France; ^24^ Bulgarian Society for the Protection of Birds Sofia Bulgaria; ^25^ Hellenic Ornithological Society Athens Greece; ^26^ The Royal Society for the Conservation of Nature (RSCN) Jubaiha Jordan; ^27^ Bird Protection and Study Society of Serbia Novi Sad Serbia; ^28^ Nase Ptice Ornithological Society Sarajevo Bosnia and Herzegovina; ^29^ Macedonian Ecological Society Skopje Macedonia; ^30^ History Museum of Montenegro, Trg Vojvode Bećir‐bega Osmanagića 16 Podgorica Montenegro; ^31^ Istituto Superiore per la Protezione e la Ricerca Ambientale (ISPRA) Ozzano dell'Emilia Italy; ^32^ Department of Biology University of Turku Turku Finland

**Keywords:** adaptation strategies, climate change, coastal wetlands, conservation planning, exposure assessment, International Waterbird Census, protected areas, Ramsar Convention, áreas protegidas, cambio climático, Censo Internacional de Aves Acuáticas, Convención Ramsar, estrategias de adaptación, evaluación de la exposición, humedales costeros, planeación de la conservación, 适应策略, 气候变化, 沿海湿地, 保护规划, 暴露风险评估, 国际水鸟普查, 保护地, 拉姆萨尔公约

## Abstract

Sea‐level rise (SLR) is expected to cause major changes to coastal wetlands, which are among the world's most vulnerable ecosystems and are critical for nonbreeding waterbirds. Because strategies for adaptation to SLR, such as nature‐based solutions and designation of protected areas, can locally reduce the negative effects of coastal flooding under SLR on coastal wetlands, it is crucial to prioritize adaptation efforts, especially for wetlands of international importance for biodiversity. We assessed the exposure of coastal wetlands important for nonbreeding waterbirds to projected SLR along the Mediterranean coasts of 8 countries by modeling future coastal flooding under 7 scenarios of SLR by 2100 (from 44‐ to 161‐cm rise) with a static inundation approach. Exposure to coastal flooding under future SLR was assessed for 938 Mediterranean coastal sites (≤30 km from the coastline) where 145 species of nonbreeding birds were monitored as part of the International Waterbird Census and for which the monitoring area was delineated by a polygon (64.3% of the coastal sites monitored in the Mediterranean region). Thirty‐four percent of sites were threatened by future SLR, even under the most optimistic scenarios. Protected study sites and study sites of international importance for waterbirds were, respectively, 1.5 and 2 times more exposed to SLR than the other sites under the most optimistic scenario. Accordingly, we advocate for the development of a prioritization scheme to be applied to these wetlands for the implementation of strategies for adaptation to SLR to anticipate the effects of coastal flooding. Our study provides major guidance for conservation planning under global change in several countries of the Mediterranean region.

## Abstract (es)

Exposición de los humedales de importancia para las aves acuáticas no reproductoras al incremento del nivel del mar en el Mediterráneo

**Resumen:**

Se espera que el incremento en el nivel del mar (INM) cause cambios importantes en los humedales costeros, los cuales se encuentran entre los ecosistemas más vulnerables y son críticos para las aves acuáticas no reproductoras. Es crucial la priorización de los esfuerzos de adaptación, especialmente en los humedales con importancia internacional para la biodiversidad, ya que las estrategias de adaptación ante el INM, como las soluciones basadas en la naturaleza y la designación de áreas protegidas, pueden reducir localmente los efectos negativos de las inundaciones costeras por INM en los humedales costeros. Evaluamos la exposición de los humedales costeros con importancia para las aves acuáticas no reproductoras ante el INM proyectado en las costas del Mediterráneo en ocho países con un modelo de inundaciones costeras en el futuro bajo siete escenarios de INM para el año 2100 (de 44 a 161 cm) con un enfoque de inundación estática. Evaluamos la exposición a las inundaciones costeras bajo el INM futuro en 938 sitios costeros del Mediterráneo (≤ 30 km a partir de la costa), en donde monitoreamos a 145 especies de aves no reproductoras como parte del Censo Internacional de Aves Acuáticas y para los cuales el área de monitoreo estuvo delineada con un polígono (64.3% de los sitios costeros monitoreados en la región Mediterránea). El 34% de los sitios se vio amenazado por el INM en el futuro, incluso con los escenarios más optimistas. Los sitios de estudio protegidos y los sitios de estudio de importancia internacional para las aves acuáticas estuvieron expuestos 1.5 y 2 veces más al INM que otros sitios con el escenario más optimista. De acuerdo con esto, abogamos por el desarrollo de un esquema de priorización para aplicarse en estos humedales para la implementación de estrategias de adaptación al INM para anticipar los efectos de las inundaciones costeras. Nuestro estudio proporciona información importante para la planeación de la conservación bajo el cambio global en varios de los países del Mediterráneo.

## 地中海非繁殖水鸟的重要湿地受海平面上升影响的情况

【摘要】 海平面上升预计将导致沿海湿地发生重大变化, 而这些湿地作为世界上最脆弱的生态系统之一, 对非繁殖水鸟至关重要。由于海平面上升的适应策略(如基于自然的解决方案和划定保护区)可以局部减少海平面上升导致的沿海洪水对沿海湿地的负面影响, 因此优先实施这些适应策略十分重要, 尤其是针对那些对生物多样性具有国际重要性的湿地。本研究用静态淹没方法模拟了至2100年七种海平面上升情景(上升44‐161厘米)下未来的沿海洪水情况, 并评估了八个国家地中海沿岸对非繁殖水鸟十分重要的沿海湿地的受影响程度。我们共评估了938个地中海沿海位点(距海岸线30公里以内)未来的沿海洪水暴露情况, 这些位点有145种受到国际水鸟普查监测的非繁殖鸟类, 监测区域被划定为多边形(占地中海地区受监测沿海位点的64.3%)。我们发现, 即使在最乐观的情况下, 也有34%的位点受未来海平面上升的威胁;受保护的研究位点和对水鸟具有国际重要性的研究位点受海平面上升影响的暴露风险分别是其他位点的1.5倍和2倍。因此, 我们提议应针对这些湿地制定优先保护方案, 实施海平面上升的适应策略, 为提前应对沿海洪水影响做好准备。我们的研究为全球变化背景下地中海地区多个国家的保护规划提供了重要指导。**【翻译:胡怡思;审校:聂永刚】**

## INTRODUCTION

Sea‐level rise (SLR) is one of the most concerning and uncertain consequences of climate change for human activities and coastal biodiversity. Future SLR threatens up to 630 million people by the end of the 21st century (Kulp & Strauss, 2019) and might have a severe detrimental impact on economic activities. Sites of great cultural and natural value are also threatened by future SLR (Reimann et al., 2018; Vousdoukas et al., 2022). Among these sites, wetlands are highly vulnerable to anthropogenic pressures that led to a major loss of these ecosystems (70% since 1900, 35% since 1970) and their associated biodiversity (Convention on Wetlands, 2021; WWF, 2020). On top of these pressures, coastal wetlands are highly threatened by SLR; more than half of them might be lost by 2100 as a result of this pressure (Blankespoor et al., 2014; Saintilan et al., 2023; Spencer et al., 2016). Coastal flooding and its effects on wetlands can be reduced by implementing strategies for adaptation to SLR, such as engineering‐based protection strategies (e.g., construction of breakwaters, dikes, or groins), strategies involving nature‐based solutions (NbS) (e.g., ecosystem restauration), and retreat strategies supported by the designation of protected areas (PAs). Therefore, identifying priority coastal wetlands to implement these strategies could help anticipate the future impacts of SLR on these invaluable ecosystems. Because the magnitude of future SLR varies greatly under future scenarios (IPCC, 2021) and because of the high uncertainty of the contribution of the Greenland and Antarctic ice sheets to SLR (Bamber et al., 2019; Edwards et al., 2021), the exploration of risks induced by SLR under various future scenarios is of major importance.

Assessing the threat of future SLR to sites of importance for waterbirds can support conservation planning for coastal wetlands. Indeed, the conservation of waterbirds, which are highly dependent on wetlands, is the focus of many international conservation policies (e.g., Ramsar Convention, African–Eurasian migratory Waterbird Agreement, European Union Birds Directive). These policies led to the establishment of a long‐term monitoring scheme of waterbirds at a large scale and the implementation in wetlands of protection and management actions that provide effective conservation of these species (Amano et al., 2018; Wauchope et al., 2022), including in a context of global change (Gaget et al., 2022, 2021). Furthermore, waterbirds are used to identify wetlands of international importance (i.e., key wetlands to preserve) as part of the Ramsar Convention on wetlands. Nevertheless, although many studies report potential detrimental effects of projected SLR on coastal birds, most studies were conducted at local or national scales to date (e.g., Hatfield et al., 2012; Ivajnšič et al., 2017; Klingbeil et al., 2021; Rosencranz et al., 2018) (but see Iwamura et al. [2014, 2013]) and were limited to a few species (often not waterbirds). As a result, it is necessary to identify wetlands of importance for waterbirds exposed to SLR at country and global scales and to consider a wide range of species.

Strategies for adaption to SLR should be implemented in Mediterranean coastal wetlands because they host an extraordinary biodiversity and are likely to be strongly affected by projected SLR (MWO‐2; Popoff et al., 2021; Schuerch et al., 2018; Spencer et al., 2016). Mediterranean wetlands and the waterbirds that use them are at risk from various anthropogenic pressures that are exacerbated in coastal areas (Galewski et al., 2021; MedECC, 2020; MWO‐2; UNEP/MAP & Plan Bleu, 2020). Worse, the Mediterranean region is expected to lose substantial wetland surface area because of SLR (Schuerch et al., 2018; Spencer et al., 2016). As a result, the future impacts of SLR on coastal wetlands have been identified as a key issue for the conservation of Mediterranean wetlands (Taylor et al., 2021). Identifying priority coastal wetlands of importance for waterbirds to implement strategies for adaptation to SLR in the Mediterranean region could help anticipate the future impacts of SLR on these ecosystems. Reinforcing PAs network, for instance, can support retreat strategies by facilitating the inland migration of wetlands. It is also especially important as major gaps in wetland protection were identified in this region (Leberger et al., 2020; Popoff et al., 2021; Verniest et al., 2023), and many new PAs will have to be designated to meet the protection targets of the Kunming–Montreal global biodiversity framework (CBD, 2022).

We identified the wetlands monitored for nonbreeding waterbirds that are most at risk from future SLR by the end of the century in 8 Mediterranean countries, with an emphasis on wetlands of international importance for waterbirds (sensu Ramsar Convention), in order to guide priorities for the implementation of adaptation measures to SLR, including wetlands to protect. To this end, we assessed the exposure (sensu Intergovernmental Panel on Climate Change [IPCC]) to coastal flooding under future SLR of 938 Mediterranean coastal wetlands for which nonbreeding waterbirds are monitored as part of the International Waterbird Census (IWC) (Delany et al., 2005) and for which the status of international importance for waterbirds was assessed in Popoff et al. (2021) (64.3% of the IWC coastal sites of the Mediterranean region). Using results from Popoff et al. (2021), we also assessed the exposure of waterbird species to SLR from the exposure of their sites of international importance. Projected flooded surface was modeled using a static inundation approach under 7 future SLR scenarios of the IPCC combining radiative forcing levels with socioeconomic conditions (Eyring et al., 2016; O'Neill et al., 2016) and ranging from 44‐ to 161‐cm of SLR by 2100 (IPCC, 2021). Our findings can inform the allocation of conservation resources to lessen the vulnerability of coastal wetlands to coastal flooding through the implementation of strategies for adaptation to SLR in 8 Mediterranean countries.

## METHODS

### Waterbird monitoring sites

We initially selected 1474 coastal sites (i.e., ≤30 km from the Mediterranean coastline [Popoff et al., 2021]) monitored for waterbirds at least once between 1993 and 2017 as part of the IWC in 20 Mediterranean countries (Appendix S1). The IWC program, coordinated by Wetlands International (http://www.wetlands.org), is conducted each year by skilled volunteers and professionals who monitor nonbreeding waterbird populations in thousands of wetlands following a standardized protocol (Delany, 2005). Following Verniest et al. (2023), sites without information on the location, sites whose location was considered imprecise, and sites that were potentially duplicates or aggregates of other sites were removed beforehand. We filtered out sites for which only the centroid and no polygon delineating the monitored area were provided (*n* = 526, 35.7%) after assessing the bias induced by their inclusion compared with sites with polygons because computing the exposure of sites to SLR from site centroids instead of polygons resulted in a strong underestimation of site exposure and this underestimation strongly differed between SLR scenarios (Appendix S2). Countries for which site polygons were completely missing were consequently not included in this study (Albania, Bosnia and Herzegovina, Cyprus, Israel, Lebanon, Malta, Montenegro, Slovenia, Turkey [Appendix S1]). Egypt, Spain, and Syria were also not considered because their number or proportion of sites with polygons was very low. Finally, because site polygons can overlap inland habitats (i.e., terrestrial habitats and inland waters) and marine habitats (as opposed to inland habitats), 6 fully marine sites were deleted because they were already completely flooded. From the initial 1474 sites, the abovementioned criteria resulted in a subset of 938 study sites (64.3%) in 8 countries (Algeria, Croatia, France, Greece, Italy, Libya, Morocco, Tunisia [Appendix S3]) with a total surface area of 10,274 km^2^.

**FIGURE 1:**
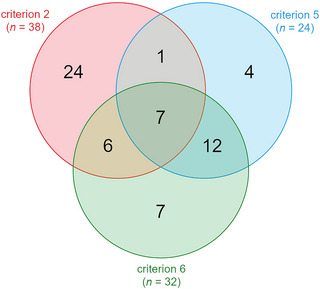
Number of study sites of international importance for waterbirds (sensu Ramsar Convention) (61 total) that regularly hosted threatened species (criterion 2), regularly hosted at least 20,000 waterbirds (criterion 5), or regularly hosted at least 1% of the individuals in a population of one species or subspecies of waterbird (criterion 6).

Sixty‐one of these sites were identified as of international importance for waterbirds (sensu Ramsar Convention) by Popoff et al. (2021), that is, sites that regularly host globally threatened species (i.e., vulnerable, endangered, or critically endangered) (criterion 2), at least 20,000 waterbirds (criterion 5), or at least 1% of the individuals in a population of one species or subspecies of waterbird (criterion 6) (Figure 1). Study sites whose status of international importance for waterbirds was not assessed by Popoff et al. (2021) (*n* = 206, 22.0%) because they were monitored <5 years and therefore could not de facto meet any criteria of international importance were not considered of international importance for waterbirds.

Study sites whose polygon overlapped a PA (*n* = 562, 59.9%) were considered protected, regardless of the level of overlap. PA boundaries were extracted from the Mediterranean Wetlands Observatory database, which combines systematic national inventories with various international sources (e.g., the World Database of Protected Areas, https://www.protectedplanet.net; Common Database on Designated Areas, https://www.eea.europa.eu; and sites designated under international agreements [e.g., the Ramsar Convention, Natura 2000]). Study sites with polygons that did not overlap a PA were referred to as unprotected sites (*n* = 376, 40.1%). Protected sites were slightly overrepresented in our sample compared with the sample of all coastal sites monitored for nonbreeding birds (with or without polygons) (Appendix S4).

### Modeling coastal flooding under SLR

We modeled coastal flooding under SLR with a hydrologically connected bathtub method (i.e., static inundation approach). This method models the sea surface by specifying a sea level and then identifying areas below the defined sea level on a digital elevation model (DEM) and connected to the sea. We only modeled coastal flooding caused by the rise of the Mediterranean Sea (sensu International Hydrographic Organization, i.e., the Sea of Marmara and the Black Sea were excluded). The workflow used to model coastal flooding is in Appendix S5.

Coastal flooding was modeled using the GLO‐90 Copernicus DEM (European Space Agency & Airbus, 2021), which provides elevation data at an approximate 90‐m horizontal resolution. This DEM has a better vertical accuracy than other available global DEMs (European Space Agency, 2021). It was preferred to the 30‐m horizontal resolution version of the Copernicus DEM (GLO‐30) due to the lack of adequate computing resources to process coastal modeling at higher horizontal spatial resolution. Although the use of such a simplified coastal flooding model and coarse‐resolution elevation data is open to criticism (e.g., Gesch, 2018), we considered that its use was justified by our intentions to assess the potential future dynamics of coastal wetlands important for waterbirds at a multicountry scale and not to discuss results for specific sites. Furthermore, coarse‐resolution global DEMs have been used in many similar studies (e.g., Blankespoor et al., 2014; Reimann et al., 2018; Schuerch et al., 2018; Vousdoukas et al., 2022, 2020; Wolff et al., 2023). Further limitations are discussed below.

### SLR scenarios

We modeled coastal flooding for 7 global projections of SLR by 2100, relative to the 1995–2014 reference period. Four of these projections are median values of SLR projected under CMIP6 scenarios (Eyring et al., 2016; O'Neill et al., 2016): 44 cm (SSP1‐2.6), 56 cm (SSP2‐4.5), 68 cm (SSP3‐7.0), and 77 cm (SSP5‐8.5) (IPCC, 2021). We also used 3 more pessimistic CMIP6 projections of SLR that cannot be ruled out (IPCC, 2021): the median value projected under the SSP5‐8.5 low‐confidence scenario (SSP5‐8.5 LC) that considers additional uncertain ice sheet processes (88 cm); the upper limit of the likely range (likelihood ≥66%) under SSP5‐8.5 (SSP5‐8.5 high) (102 cm); and the 83rd percentile of the SSP5‐8.5 low confidence (SSP5‐8.5 LC high) (161 cm) (IPCC, 2021). The 83rd percentile was used instead of the upper limit of the likely range for this scenario due to the high uncertainty of some of its processes (IPCC, 2021). Given the reference dates of the various sources used to produce the vertical reference datum of the GLO‐90 Copernicus DEM (i.e., earth gravitational model 2008 [Pavlis et al., 2012]), we considered the 0‐m elevation height of this DEM to be the 0‐m elevation height of the 1995–2014 reference period, which we assumed to be a good approximation considering average current SLR (i.e., 3.7 mm/year [IPCC, 2021]), and used it to model current marine surface. We also considered that overlooking tide‐related differences in sea level was an adequate approach, given the vertical accuracy of the DEM and the very low tidal range of the Mediterranean Sea except in a few locations (e.g., Gulf of Gabes in Tunisia, northern Adriatic Sea in Italy). Finally, we assumed that SLR in the Mediterranean will be similar to SLR at global scale because the former will be driven by the Atlantic Ocean (Ali et al., 2022; Tsimplis et al., 2013).

### Site exposure to SLR

**FIGURE 2:**
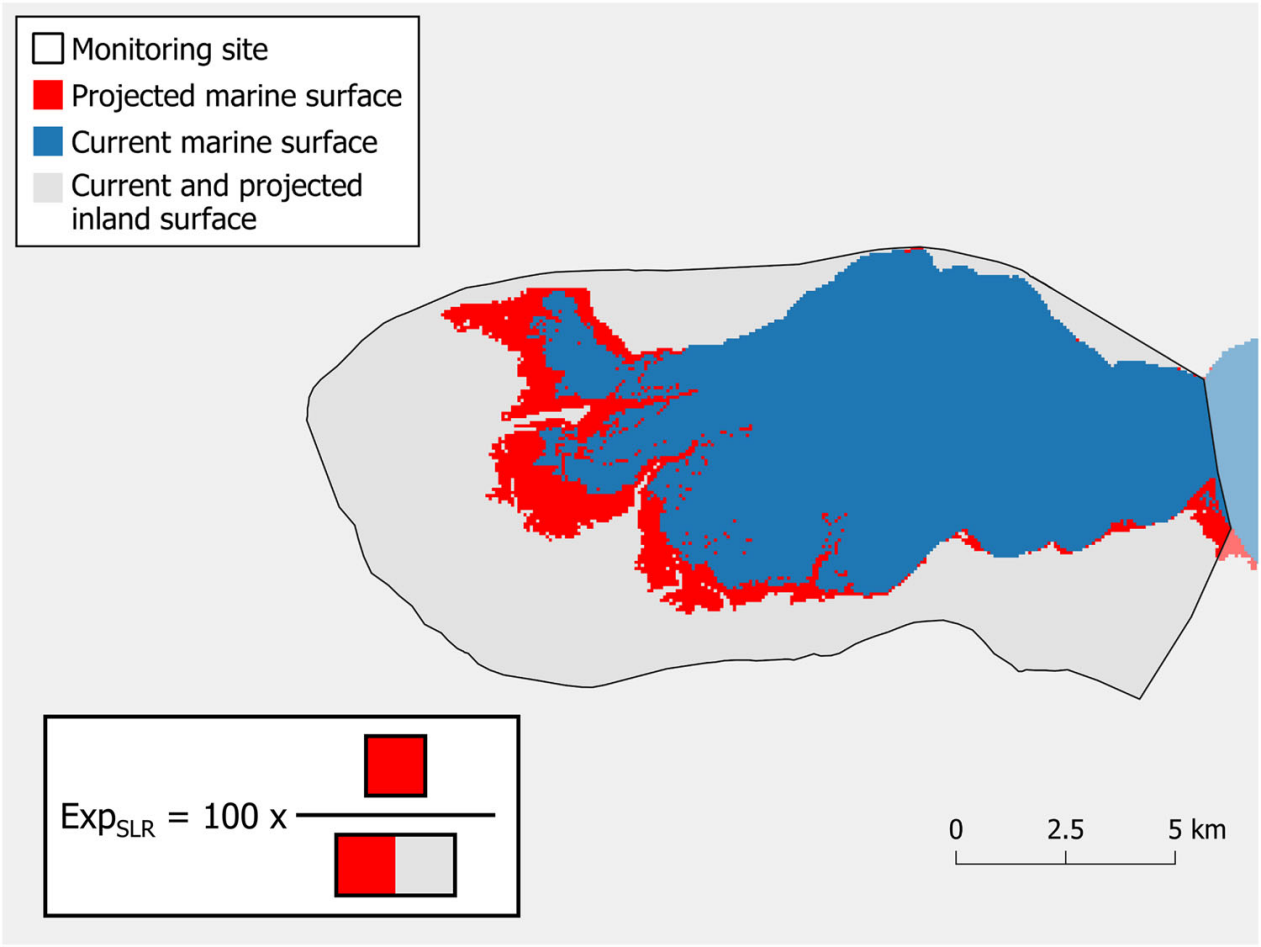
Calculation of the exposure of sites monitored for nonbreeding waterbirds to coastal flooding under sea‐level rise projected by 2100 (Exp_SLR_) applied to the Spercheios delta site (Greece) and a sea‐level rise of 77 cm (SSP5‐8.5) (Exp_SLR_ = 17.0%).

For each scenario, we computed the exposure of a study site to coastal flooding under future SLR (hereafter Exp_SLR_) as the percentage of current inland surface of the site (i.e., current marine surface of a site excluded) projected to be flooded by the sea (Equation 1; Figure 2). This exposure metric thus reflects the potential conversion of coastal and inland wetland habitats, such as shores, coastal lagoons, peatlands, salt and freshwater marshes, rivers, lakes, and even artificial wetlands (e.g., salines, aquaculture ponds, seasonally flooded agriculture lands), into permanent shallow marine waters or estuarine waters.

(1) $$\mathrm{Ex}p_{\mathrm{SLR}}=100\times \frac{a_{i}}{a_{i}+b_{i}},$$

where *a_i_* is the projected sea surface (i.e., current inland surface area projected to be flooded [red in Figure 2]) at site *i* and *b_i_* is the current and projected inland surface at site *i* (gray in Figure 2). Note that Exp_SLR_ was also computed for study sites that are partially marine. We considered a site exposed to SLR when Exp_SLR_ > 0%.

### Data analyses

We compared the exposure to coastal flooding under SLR between protected and unprotected study sites and between study sites of international importance for waterbirds and sites not of international importance for waterbirds with beta regressions (Ferrari & Cribari‐Neto, 2004) fitted with the glmmTMB package (Brooks et al., 2017) after transforming Exp_SLR_ following Smithson and Verkuilen (2006). Statistical significance was assessed using 95% confidence intervals.

We also assessed the exposure of species to SLR at their sites of international importance by assessing, for each species that caused at least one study site to meet criterion 2 (regularly hosting threatened species) (*n* = 6) or criterion 6 (regularly hosting at least 1% of the individuals in a population of one species or subspecies) (*n* = 25), the number and average Exp_SLR_ of exposed sites for which the species caused it to be considered of international importance.

Geoprocessing operations to model coastal flooding were performed using ArcGIS 10.8 (ESRI, 2019) and the spatial analyst extension. Statistical analyses and figure production were performed using R 4.1.0 (R Core Team, 2021). Maps were generated using QGIS 3.4.15 (QGIS, Development Team, 2020).

## RESULTS

### Site exposure to SLR

**FIGURE 3:**
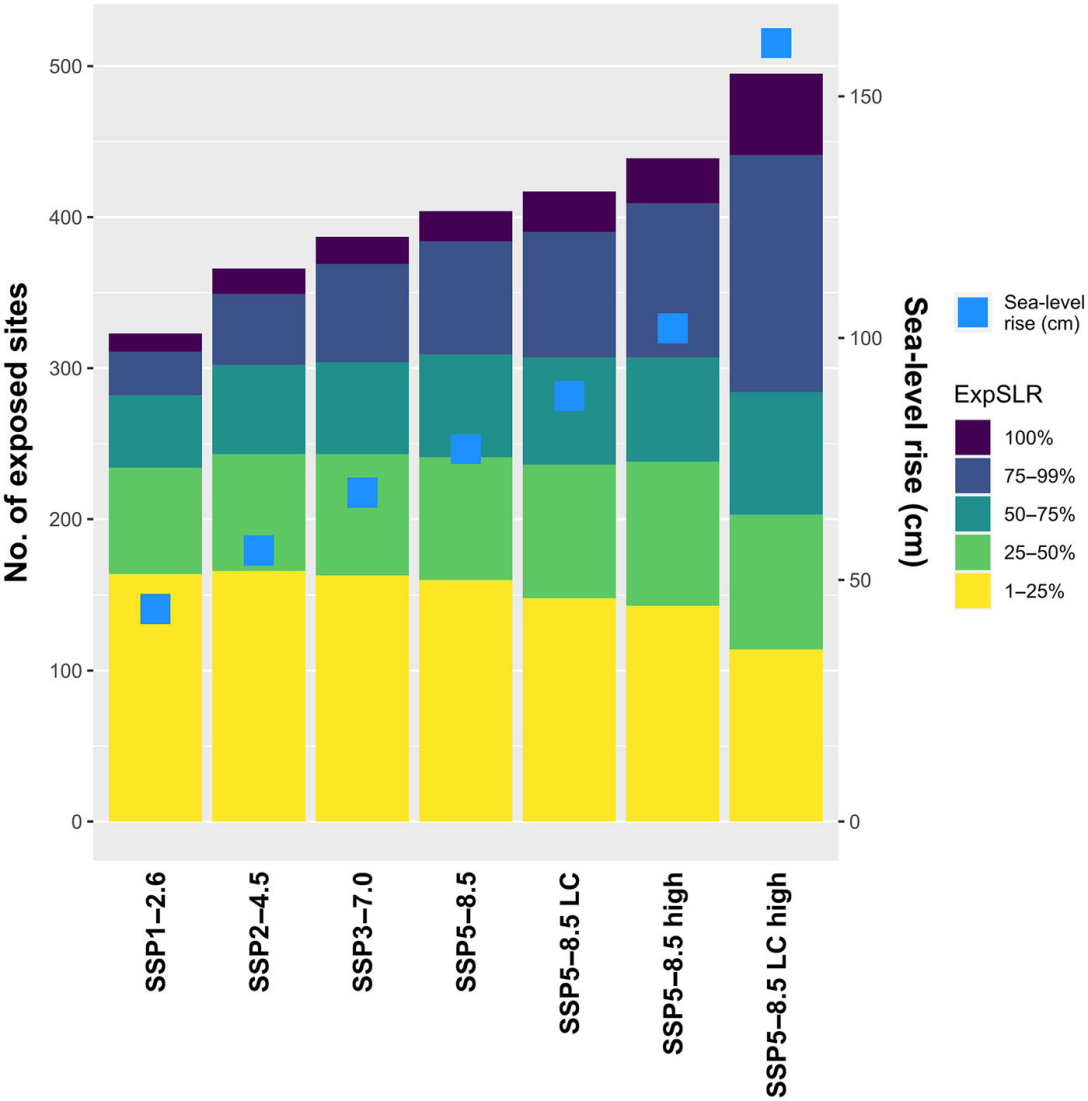
Number of study sites monitored for nonbreeding waterbirds exposed to projected sea‐level rise (SLR) in the Mediterranean by 2100 (left axis) and projected sea‐level rise (blue squares, right axis) under each scenario (LC, low‐confidence scenario that considers additional uncertain ice sheet processes; high, upper limit of the likely range for SSP5‐8.5 and 83rd percentile for SSP5‐8.5 LC). A map of these sites for SSP1‐2.6 and SSP5‐8.5 LC high is in Figure 4.

**FIGURE 4:**
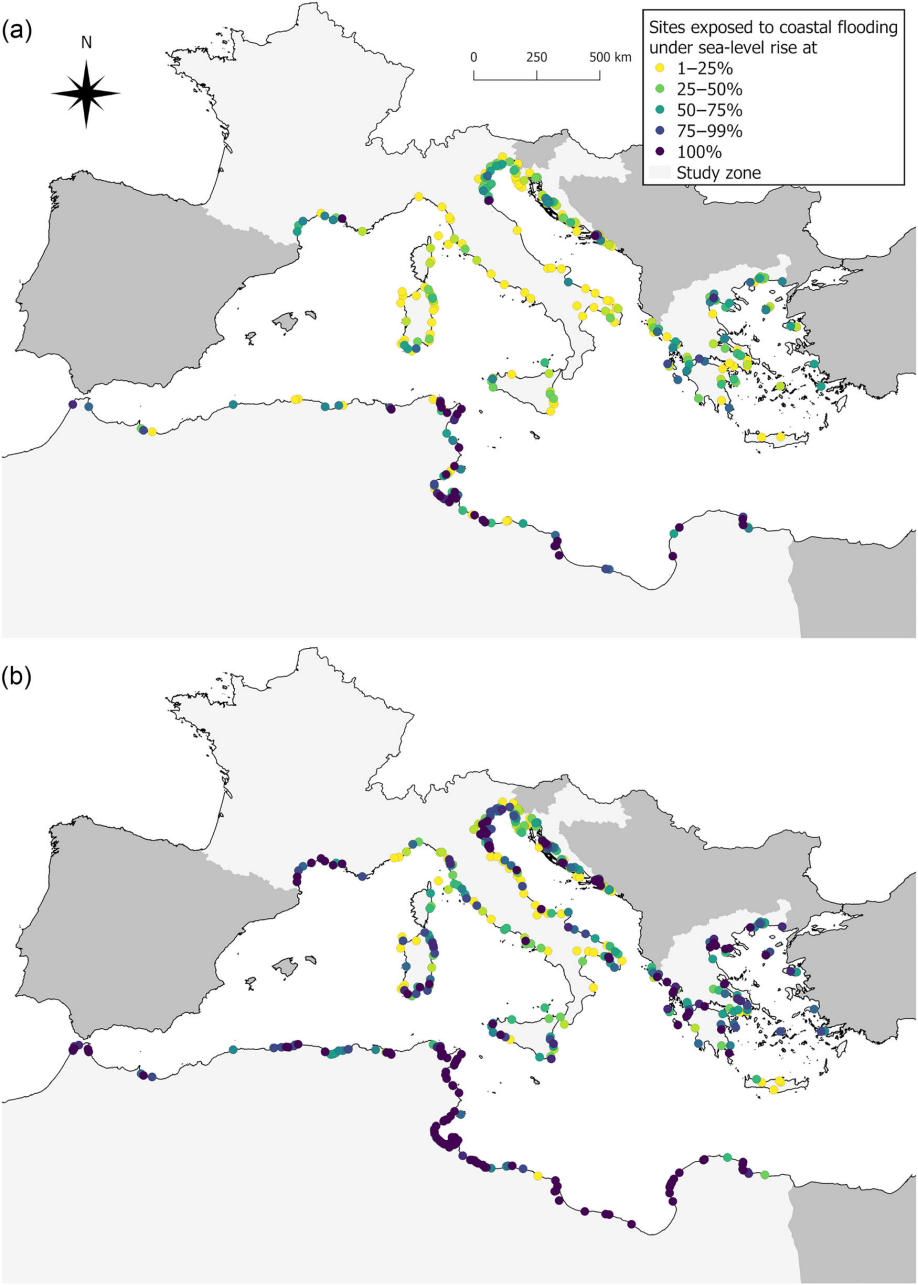
Study sites monitored for nonbreeding waterbirds that are exposed to projected sea‐level rise (Exp_SLR_ > 0%) in the Mediterranean by 2100 under (a) SSP1‐2.6 and (b) SSP5‐8.5 LC high (83rd percentile of the SSP5‐8.5 scenario that considers additional uncertain ice sheet processes) (light gray, the 8 countries considered in this study [Algeria, Croatia, France, Greece, Italy, Libya, Morocco, Tunisia]; dark gray, countries in which the International Waterbird Census is carried out but for which no site was selected).

Among the 938 study sites, 323 (34.4%, SSP1‐2.6) to 495 (52.8%, SSP5‐8.5 LC high) were exposed to projected SLR by 2100 (Exp_SLR_ > 0%) (Figure 3). A list of these sites is in Appendix S10. The average Exp_SLR_ values (i.e., percentage of current inland surface of the site projected to be flooded by the sea) ranged from mean (SD) 11.41% (23.89) (SSP1‐2.6) to 31.34% (38.70) (SSP5‐8.5 LC high) according to future SLR scenarios, corresponding to 859.8 and 1901.7 km^2^ of additional sea surface area by 2100, respectively. From 12 (SSP1‐2.6) to 54 study sites (SSP5‐8.5 LC high) were expected to have their inland area fully flooded by the sea by 2100 (i.e., Exp_SLR_ = 100%), including 5 to 18 sites that currently have no marine part, respectively. The most exposed study sites were in Tunisia and Libya (Figure 4).

### Exposure of sites of international importance for waterbirds to SLR

**FIGURE 5:**
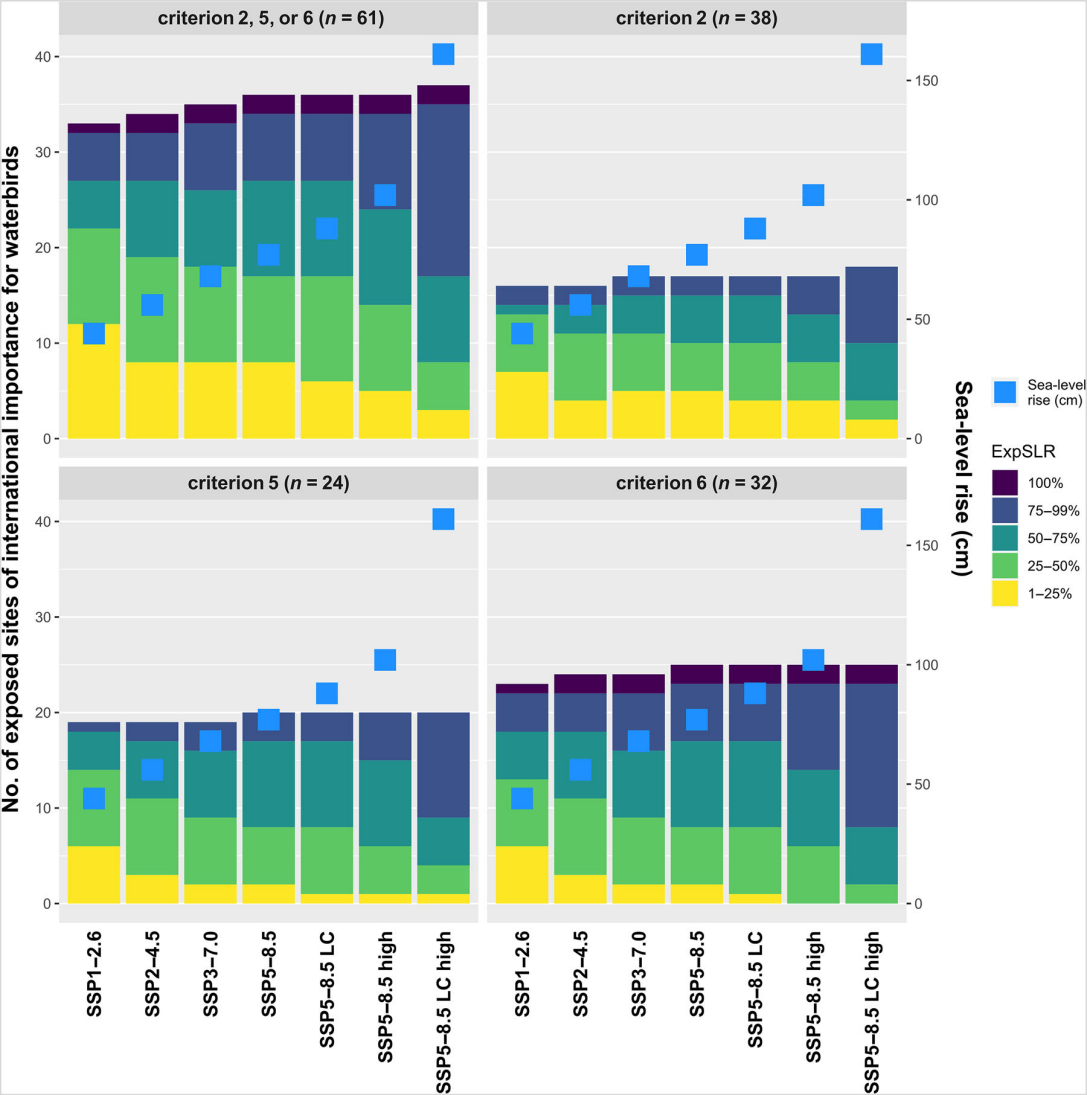
Number of study sites of international importance for waterbirds exposed to projected sea‐level rise in the Mediterranean by 2100 (left axis) and projected sea‐level rise (blue squares, right axis) per Ramsar criterion and scenario (criterion 2, site that regularly hosted threatened species; criterion 5, site that regularly hosted at least 20,000 waterbirds; criterion 6, site that regularly hosted at least 1% of individuals in a population of one species or subspecies of waterbird; LC, low‐confidence scenario that considers additional uncertain ice sheet processes; high, upper limit of the likely range for SSP5‐8.5 and 83rd percentile for SSP5‐8.5 LC). A map of these sites is in Figure 6 for SSP5‐8.5 LC high and in Appendix S6 for SSP1‐2.6.

**FIGURE 6:**
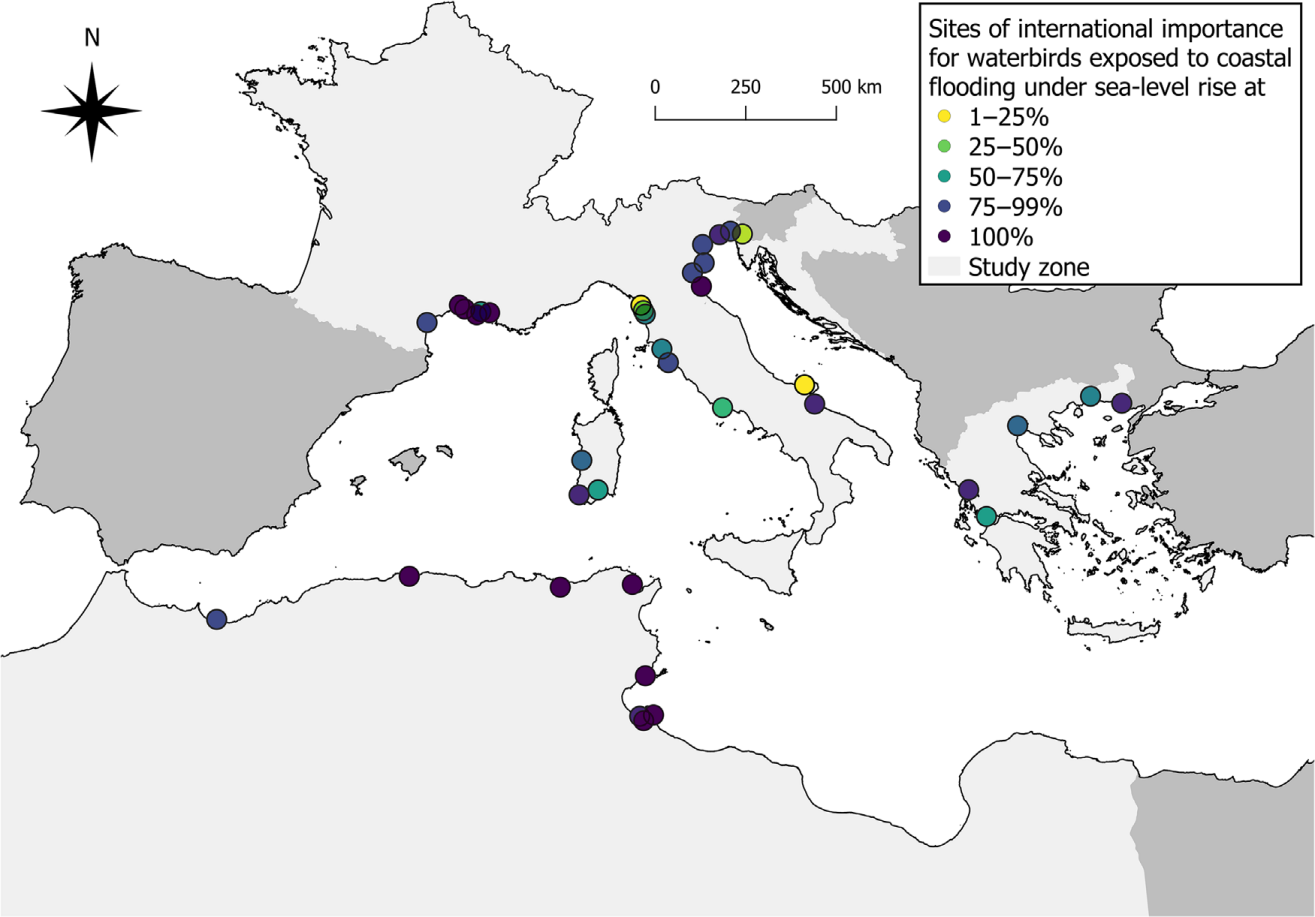
Study sites of international importance for waterbirds that are exposed to projected sea‐level rise (Exp_SLR_ > 0%) in the Mediterranean by 2100 under SSP5‐8.5 LC high (83rd percentile of the SSP5‐8.5 scenario that considers additional uncertain ice sheet processes) (light gray, 8 countries considered in this study [Algeria, Croatia, France, Greece, Italy, Libya, Morocco, Tunisia]; dark gray, Mediterranean countries in which the International Waterbird Census is carried out but for which no site was selected). Map of study sites of international importance for waterbirds that are exposed to projected sea‐level rise in the Mediterranean by 2100 under SSP1‐2.6 is in Appendix S6.

Among the 61 study sites of international importance for waterbirds, 33 (54.1%, SSP1‐2.6) to 37 sites (60.7%, SSP5‐8.5 LC high) were exposed to projected SLR by 2100 (Exp_SLR_ > 0%) (Figures 5 & 6; Appendix S6), and only one was not protected. Study sites of international importance for waterbirds were overall more exposed to projected SLR than sites not of international importance for waterbirds (Appendix S7). The average Exp_SLR_ ranged from mean (SD) 21.55% (30.81) (SSP1‐2.6) to 43.08% (40.76) (SSP5‐8.5 LC high) at these sites, whereas at sites not of international importance, the average Exp_SLR_ ranged from 10.71% (23.19) (SSP1‐2.6) to 30.52% (38.45) (SSP5‐8.5 LC high). Almost 80% of study sites of international importance for waterbirds exposed to projected SLR (*n* = 29) were expected to have half of their inland area flooded by the sea by 2100 (i.e., Exp_SLR_ > 50%) under SSP5‐8.5 LC high (Figure 5). Study sites regularly hosting at least 20,000 waterbirds (Ramsar criterion 5) or at least 1% of the individuals in a population of one species or subspecies of waterbird (Ramsar criterion 6) were particularly exposed to projected SLR, with more than 75% of these sites exposed in the worst‐case scenario (83.3%, *n* = 20 and 78.1%, *n* = 25, respectively) (Figure 5).

### Exposure of protected sites to SLR

Among the 562 protected study sites, 245 (43.6%, SSP1‐2.6) to 349 (62.1%, SSP5‐8.5 LC high) were exposed to projected SLR by 2100 (Exp_SLR_ > 0%) (Appendix S8). Consequently, only 78 (SSP1‐2.6) to 146 unprotected sites (SSP5‐8.5 LC high) were exposed to projected SLR (i.e., Exp_SLR_ > 0%). Protected study sites were more exposed than unprotected sites (Appendix S9), with average Exp_SLR_ values ranging from mean (SD) 13.01% (23.37) (SSP1‐2.6) to 34.71% (38.16) (SSP5‐8.5 LC high) at protected sites, whereas average Exp_SLR_ values at unprotected sites ranged from 9.02% (24.48) (SSP1‐2.6) to 26.30% (39.01) (SSP5‐8.5 LC high). Almost 200 protected study sites (*n* = 192) were expected to have half of their inland area flooded by the sea by 2100 (i.e., Exp_SLR_ > 50%) under SSP5‐8.5 LC high.

### Exposure of species to SLR at their sites of international importance

**FIGURE 7:**
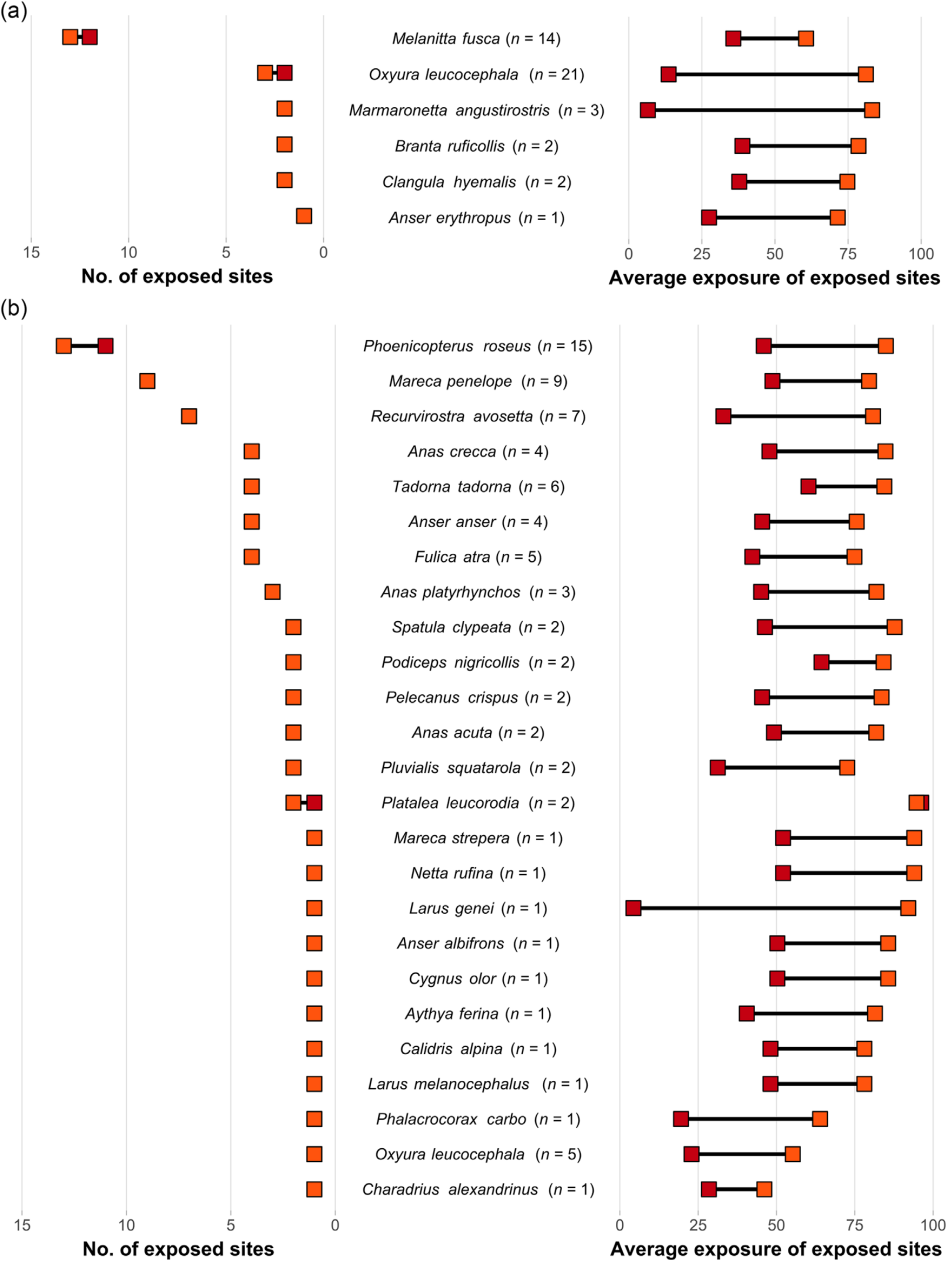
Number (left) and average exposure (right) of study sites of international importance for waterbirds that meet (a) Ramsar criterion 2 (regularly hosted threatened [i.e., vulnerable, endangered, or critically endangered] species) or (b) Ramsar criterion 6 (regularly hosted at least 1% of the individuals in a population of one species or subspecies of waterbird) that are exposed to projected sea‐level rise in the Mediterranean by 2100 under SSP1‐2.6 (red) and SSP5‐8.5 LC high (orange) per species meeting criteria 2 (*n* = 6) or 6 (*n* = 25) (number in parentheses, total number of sites of international importance per species).

All 6 threatened species that caused a study site to meet criterion 2 had at least one site of international importance exposed to projected SLR, even under SSP1‐2.6 (Figure 7). The velvet scoter (*Melanitta fusca*), which was the second highest contributing species to sites meeting criterion 2 (*n* = 14 out of 38 study sites), had the highest number of exposed sites of international importance by far. The red‐breasted goose (*Branta ruficollis*), the long‐tailed duck (*Clangula hyemalis*), and the lesser white‐fronted goose (*Anser erythropus*) contributed to few sites meeting criterion 2 (*n* = 2, 2, and 1, respectively), but all of their study sites of international importance were exposed to projected SLR and at high levels of exposure. Although the white‐headed duck (*Oxyura leucocephala*) was the species that contributed the most to sites meeting criterion 2 (*n* = 21 out of 38 study sites), few of its sites of international importance were exposed in the 8 countries considered and their exposure varied widely between scenarios (Figure 7).

All 25 species that caused a study site to meet criterion 6 had at least one site of international importance exposed to projected SLR, including 13 *Anatidae* species (Figure 7). The 3 highest contributing species to sites meeting criterion 6, the greater flamingo (*Phoenicopterus roseus*), the Eurasian wigeon (*Mareca penelope*), and the pied avocet (*Recurvirostra avosetta*) (*n* = 15, 9, and 7 out of 32 study sites, respectively), had the highest number of exposed sites of international importance in the 8 countries considered. For most species, nearly all study sites of international importance were exposed to projected SLR. The number of exposed sites under SSP1‐2.6 was overall very similar to the number of exposed sites under SSP5‐8.5 LC high (Figure 7).

## DISCUSSION

Coastal wetlands, which are critical ecosystems for nonbreeding waterbirds in the Mediterranean region (Popoff et al., 2021), are highly threatened by various anthropogenic pressures, not least of which is SLR (Schuerch et al., 2018; Spencer et al., 2016). Our results, which are centered on coastal waterbird communities, can guide the prioritization of coastal wetlands for the implementation of adaptation strategies to mitigate their vulnerability to future SLR in the 8 countries considered.

### Limitations of our framework

The number of sites and the area exposed to coastal flooding under SLR was likely underestimated. First, we were able to perform the analyses on only two thirds of Mediterranean coastal sites (64.3%) (those for which the monitoring areas are delineated by polygons), leading to the exclusion of entire countries (e.g., Spain) and 526 (35.7%) coastal sites around the Mediterranean Sea. As a result, we estimated that we may have omitted up to 200 (29.4%) exposed IWC sites (Appendix S2). Nevertheless, this exclusion also ensured a better estimate of the exposure of study sites to SLR. We stress the need to provide support to IWC national coordinators to ensure that each site is assigned a polygon following the standard methods developed by Wetlands International (2016). This would enable the comprehensive identification of sites exposed to local anthropogenic pressures, such as SLR, in all Mediterranean countries and to consider them in prioritization schemes for implementing conservation measures. The lack of spatial information on IWC monitoring sites is currently one of the main limitations to conducting conservation studies on nonbreeding waterbirds in the western Palearctic.

In addition, the hydrologically connected bathtub method we used has several limitations that may have led to uncertainties in our assessment of exposure to SLR. Indeed, this approach models coastal flooding without considering important hydrological and geomorphological processes, such as seasonal high waves, coastal erosion, groundwater flooding, and salinization, thereby potentially underestimating the land area exposed to projected SLR by up to one‐half (Anderson et al., 2018). For instance, salinization, which is mainly driven in coastal wetlands by surface or subsurface seawater intrusion (Mazhar et al., 2022), can strongly alter geochemical processes of Mediterranean coastal wetlands and affect their biological communities (e.g., zooplankton, macroinvertebrates, aquatic plants) (Martínez‐Megías & Rico, 2022). Although these processes contribute substantially to the impacts of SLR on wetland ecosystems, incorporating them into coastal flood modeling is not only a methodological challenge, but is above all limited by data availability in studies at this spatial scale. Combined with the noninclusion of subsidence, also due to a lack of available data, study sites identified as being at low or no exposure to SLR and associated waterbird communities might still be exposed, especially under extreme events and natural variations (Reynolds et al., 2015).

In contrast, interpreting the surface flooded by the sea as a proxy for the net loss of inland habitats to marine habitats most likely overestimates this loss. Indeed, the static inundation approach of the hydrologically connected bathtub method does not consider hydrological and geomorphological parameters, such as sedimentation processes (e.g., vertical accretion) and their potential response to SLR, contrary to dynamic response models (Schuerch et al., 2018). Consequently, we did not model transitions between habitat types, and especially inland migration of wetlands. Therefore, we may have overestimated the exposure of coastal wetlands and waterbirds to SLR. In addition, the engineering structures already implemented to protect against SLR were overlooked. However, we assumed that these limitations do not undermine the relevance of this proxy within the framework of our study because the accommodation space available for the inland migration of wetlands might be very constrained in the highly urbanized Mediterranean coasts (MedECC, 2020); the sediments required for vertical accretion may not be sufficient in the Mediterranean region (Schuerch et al., 2018) due to the low tidal range and river management (Day et al., 1995); and current protection measures against SLR might be ineffective in high SLR scenarios if not reinforced. Inland wetland migration and transitions between habitat types can be modeled with dynamic response models (e.g., the sea level affecting marshes model [SLAMM] [Clough et al., 2010]) that were used in local‐ or country‐scale studies (e.g., Hatfield et al., 2012; Klingbeil et al., 2021; Sims et al., 2013). Although these models are more realistic than static inundation approaches because they include more hydrological and geomorphological processes (Anderson et al., 2018), they require many different fine‐scale data sets: high‐resolution elevation data (e.g., lidar, bathymetry), hard engineering structures, land cover, hydrological, geomorphological, and socioeconomic parameters, and so forth. This data‐intensive nature of dynamic response models of coastal flooding results in their application to only a few study sites most of the time and is the main reason we did not use them. Although the development of such data at global scale would enable the use of dynamic response models at larger scales, high‐resolution high‐accuracy global elevation data would already improve projections of future coastal flooding at large scale with static inundation approaches (Gesch, 2018).

The status of international importance for waterbirds of study sites might be different by 2100, due to changes in conservation status of species (affecting Ramsar criterion 2), temporal trends of waterbird populations (affecting Ramsar criterion 6), or more importantly due to distribution shifts caused by climate change (Hole et al., 2009; Johnston et al., 2013; Pavón‐Jordán et al., 2019) (affecting Ramsar criteria 2, 5, and 6). In addition, our study was limited to the international importance for nonbreeding waterbirds, whereas SLR could have significant detrimental effects on waterbird populations in the breeding and the migration periods (Hatfield et al., 2012; Ivajnšič et al., 2017; Iwamura et al., 2014, 2013; Reynolds et al., 2015).

### Implications for waterbird conservation

More than half of coastal sites of international importance for waterbirds (sensu Ramsar Convention) located in the 8 Mediterranean countries we examined could lose part of their inland habitats to coastal flooding under SLR by 2100, even under the most optimistic scenario (i.e., SSP1‐2.6: +44 cm), and were overall more exposed than coastal sites not of international importance for waterbirds. The high exposure of these sites, which provide specific habitats (e.g., coastal lagoons, salt marshes) to numerous waterbird species that often have limited inland alternative habitats, could have negative effects on Mediterranean waterbird populations and on many other taxa and ecosystem services. Therefore, we advocate for the most urgent implementation of adaptation measures in sites of international importance for waterbirds exposed to coastal flooding under future SLR.

Various strategies can be adopted to reduce or prevent coastal flooding and mitigate its effects on coastal wetlands (see Bongarts Lebbe et al., 2021; Brito & Naia, 2020): engineering‐based protection strategies, strategies involving NbS, and retreat strategies. Protection strategies based on soft engineering actions (e.g., beach nourishment) or hard engineering actions (e.g., construction of breakwaters, dikes, or groins), although effective, are short term and expensive. As a result, they are usually only implemented to protect coastal areas with high socioeconomic value. NbS, such as restoring ecosystems and allowing the natural dynamics of the coast, can also effectively address the impacts of SLR (Chausson et al., 2020; Sánchez‐Arcilla et al., 2022) while favoring biodiversity, including waterbirds (Baptist et al., 2021; Moraes et al., 2022), and providing many ecosystem services (Monty et al., 2016). However, NbS might not be appropriate in all situations, and decision makers are less familiar with NbS than engineering‐based strategies (Loizidou et al., 2023). Finally, retreat strategies can be an alternative in some contexts, although in specific situations this strategy might not be well accepted by local stakeholders and decision makers (Loizidou et al., 2023) and inland migration of wetlands might be hindered by natural or anthropogenic barriers. In addition, newly migrated wetlands may initially have lower habitat quality for birds than the original wetlands (Taillie & Moorman, 2019).

These strategies for adaptation to SLR and hybrid approaches combining multiple strategies have already been implemented in several sites that we identified as exposed to coastal flooding under future SLR. For instance, measures of dune fixation with vegetation (*Eucalyptus* and *Acacias*) have been implemented in the mouth of the Moulouya River in Morocco (Laouina, 2010; Qarro, 2012), a wetland of international importance for waterbirds (criterion 2 is met for the Marbled Teal [*Marmaronetta angustirostris*] [Popoff et al., 2021]) identified as part of the Ramsar Convention on wetlands and threatened by SLR (erosion, saltwater intrusion, habitat loss) (Dakki et al., 2003). In the Camargue (Rhône delta, France), one of the largest deltas in the Mediterranean region, the wetlands complex is considered of international importance (criterion 5 met, criterion 6 is met for many waterbird species [Popoff et al., 2021]), and hard engineering structures have been used and are still reinforced to protect the city of Saintes‐Maries de la Mer from SLR, whereas several sea dikes are no longer maintained in other areas of the delta (Puigserver et al., 2023) as part of a retreat strategy and implementation of NbS (https://rest‐coast.eu/). Conservationists and coastal managers could learn from these cases to select and implement the best strategy for adaptation to SLR in sites that are exposed to projected SLR.

More than 70% of study sites exposed to coastal flooding under SLR by 2100 overlapped PAs (SSP1‐2.6 75.9%, SSP5‐8.5 LC high 70.5%) in the 8 Mediterranean countries examined, and protected study sites were more exposed than unprotected coastal sites in all scenarios (up to 1.5 times more under the most optimistic scenario). This result was expected because many PAs were designated in coastal areas to lessen their high exposure to anthropogenic pressures (Williams et al., 2022). The implementation of the adaptation strategies mentioned above should be considered in these protected wetlands to lessen the impacts of projected SLR. We also advocate for investigating the relevance of designating new PAs in unprotected sites exposed to coastal flooding and expanding PAs in sites exposed to coastal flooding that we considered protected but do not completely overlap PAs. Indeed, the surface area of PAs needs to be increased to meet new protection targets (CBD, 2022), and PAs are a key element of retreat strategies because they can facilitate inland migration of wetlands (Brito & Naia, 2020) by reducing urbanization (Donnelly & Rodríguez‐Rodríguez, 2022) and buffer the impacts of land‐use change on newly migrated wetlands (Arizaga et al., 2020). The implementation of NbS can also be facilitated in PAs, for example, through improved access to human and economic resources, although this is not universally true because their implementation may sometimes fail to comply with the regulations or conservation objectives of PAs.

Study sites of international importance for waterbirds were up to 2 times more exposed to SLR than study sites not of international importance for waterbirds under the most optimistic scenario. Consequently, we recommend prioritizing wetlands of international importance for waterbirds exposed to coastal flooding based on other criteria to identify high‐priority targets for the implementation of strategies for adaptation to SLR.

Among globally threatened waterbird species, only 2 spend the nonbreeding season in significant numbers in the 8 Mediterranean countries we considered: the white‐headed duck and the marbled teal. The exposure to SLR of study sites of international importance for these species differed greatly among scenarios, reinforcing the need for a drastic reduction in greenhouse gas emissions, as did the difference of 172 study sites and 1041.9 km^2^ exposed to coastal flooding under SLR by 2100 between the most optimistic (SSP1‐2.6 44 cm) and worst‐case scenarios of SLR (SSP5‐8.5 LC high 161 cm).

As expected, among species for which study sites that regularly hosted at least 1% of the individuals in a population of one species or subspecies (criterion 6) were highly exposed to projected SLR, we found species strongly associated with coastal lagoons, such as the greater flamingo, the pied avocet, and the common shelduck (*Tadorna tadorna*). Several species associated with freshwater habitats were also among the most exposed species to SLR (e.g., Eurasian coot [*Fulica atra*], gadwall [*Mareca strepera*], red‐crested pochard [*Netta rufina*]) because coastal wetlands of international importance include several deltas and estuaries (e.g., Camargue, Po delta). This highlights that not all species will be affected to the same extent by coastal flooding. The effect will depend on their ecological traits and especially their association with physicochemical variables, such as salinity and water level. For instance, one can speculate that marine species, such as the Mediterranean gull (*Ichthyaetus melanocephalus*), might be affected to a lesser extent by the conversion of inland and coastal habitats to marine habitats during the nonbreeding period. Similarly, a few key areas becoming unsuitable for waterbirds due to the loss of inland habitats could have devastating effects on waterbird populations (Iwamura et al., 2013; Myers et al., 1987).

### Perspectives

Considering waterbird sensitivity to SLR alongside their exposure to this pressure (e.g., Reynolds et al., 2015) based on functional traits (e.g., salinity tolerance, preferred water level) and a trait‐based climate change vulnerability assessment framework (CCVA) (Pacifici et al., 2015) would provide valuable additional information. Quantifying species association with coastal habitats (e.g., Duan et al., 2022) with current land cover data of high spatial and thematic resolution could provide a good proxy for their sensitivity to SLR. We also recommend assessing future impacts of SLR use exposure metrics complementary to the ones we used, such as changes in water depth, which is a key factor for waterbirds (Tavares et al., 2015).

Although it is necessary to consider the potential future impacts of SLR on waterbird communities when defining priorities for the implementation of strategies for adaptation to SLR in coastal wetlands, it is imperative to also reckon with other elements we did not address. For instance, because conservation measures lack human and economic resources, especially the management of PAs (Appleton et al., 2022; Coad et al., 2019), it is also crucial to assess the cost‐effectiveness of the adaptation strategies to be implemented in the sites we identified (e.g., Iwamura et al., 2014; Luby et al., 2022). The local socioeconomic context and the national governance and regulations should also be considered to facilitate the implementation of strategies for adaptation to SLR and ensure their effectiveness (Amano et al., 2018; Obura et al., 2021).

The use of current—and eventually future—land‐cover data with high spatial and thematic resolution combined with the development of high‐resolution high‐accuracy global elevation models and flooding models would also enable projection of future coastal waterbird communities (e.g., Ivajnšič et al., 2017; Moon et al., 2021; Rosencranz et al., 2018) based on species distribution models (Convertino et al., 2012; Nuse et al., 2015) at large scale to identify potential set back sites and future sites of international importance for waterbirds. This approach would also enable assessment of the relevance of and opportunities for designating new PAs to enable retreat strategies (e.g., Tamura et al., 2023; Wolff et al., 2023). Wolff et al.’s (2020) database, for example, could be used for this.

Adapting coastal areas to SLR requires a range of risks to be considered simultaneously, including submersion, salinization (Daliakopoulos et al., 2016), and loss of biodiversity, of which bird communities are only one component. However, this component is currently not a priority when implementing strategies for adaptation to SLR because submersion and salinization, with their potential consequences for the safety of human populations, and human access to water and its various uses are often the main threats guiding the selection of these strategies. A future challenge is to integrate the threats to bird communities, and more generally to biodiversity, into the process of selecting strategies for adaptation to SLR, for instance with the implementation of NbS as an alternative or complement to traditional engineering‐based protection strategies (Sánchez‐Arcilla et al., 2022).

## Supporting information

Supporting Information

Supporting Information
