# Supplementary material for: Exposure of wetlands important for nonbreeding waterbirds to sea‐level rise in the Mediterranean
Source: Conserv Biol. 2024 May 16;38(6):e14288. doi: 10.1111/cobi.14288 (PMC11588984; doi:10.1111/cobi.14288)
Supplement: Supplementary file 1 — Supporting Information [file COBI-38-e14288-s002.docx]

**SUPPORTING INFORMATION**

Appendix S1: International Waterbird Census (IWC) coastal sites (total number, number with polygons and percentage with polygons) per country.

| **Country** | **No. of sites** | **No. of sites with polygon** | **% of sites with polygon** |
| --- | --- | --- | --- |
| Albania | 9 | 0 | 0 |
| Algeria | 165 | 119 | 72.1 |
| Bosnia and Herzegovina | 24 | 0 | 0 |
| Croatia | 132 | 128 | 97.0 |
| Cyprus | 41 | 0 | 0 |
| Egypt | 6 | 1 | 16.7 |
| France | 24 | 15 | 62.5 |
| Greece | 142 | 134 | 94.4 |
| Israel | 5 | 0 | 0 |
| Italy | 394 | 346 | 87.8 |
| Lebanon | 5 | 0 | 0 |
| Libya | 94 | 80 | 85.1 |
| Malta | 19 | 0 | 0 |
| Montenegro | 16 | 0 | 0 |
| Morocco | 28 | 24 | 85.7 |
| Slovenia | 13 | 0 | 0 |
| Spain | 164 | 2 | 1.2 |
| Syria | 1 | 1 | 100 |
| Tunisia | 155 | 98 | 63.2 |
| Turkey | 37 | 0 | 0 |
| **Total** | **1474** | **948** | **64.3** |

Appendix S2: Assessment of the bias induced by the use of the centroid coordinates of a site instead of its polygon. We compared the number of sites with polygons exposed to sea-level rise by 2100 per scenario when using the polygon and when using the centroid coordinates. From this comparison, we then estimated the number of sites with no polygon potentially exposed to sea-level rise by 2100 per scenario.

|  | **SSP1-2.6** | **SSP2-4.5** | **SSP3-7.0** | **SSP5-8.5** | **SSP5-8.5 LC** | **SSP5-8.5 high** | **SSP5-8.5 LC high** |
| --- | --- | --- | --- | --- | --- | --- | --- |
| No. of sites with polygons (n = 938) exposed |  |  |  |  |  |  |  |
| *using the polygon* | 323 | 366 | 387 | 404 | 417 | 439 | 495 |
| *using the centroid coordinates* | 46 | 61 | 71 | 77 | 84 | 96 | 142 |
| Underestimation coefficient | 7.02 | 6.00 | 5.45 | 5.25 | 4.96 | 4.57 | 3.49 |
| No. of sites with no polygon (n = 525) exposed |  |  |  |  |  |  |  |
| *measured* | 18 | 23 | 30 | 32 | 33 | 38 | 59 |
| *estimated* | 126.4 | 138.0 | 163.5 | 168.0 | 163.7 | 173.7 | 205.9 |


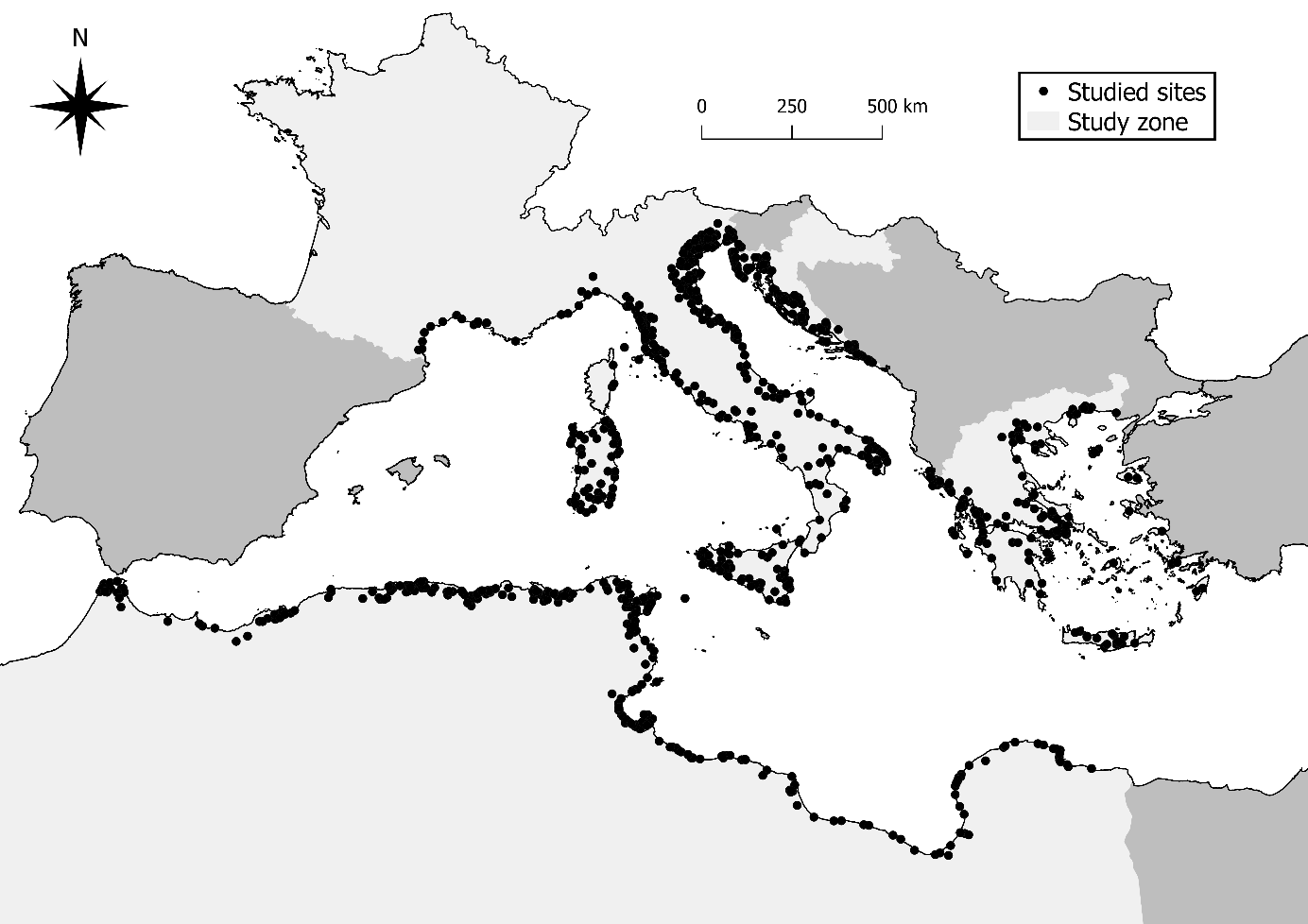


Appendix S3: Map of sites monitored as part of the International Waterbird Census (IWC) with polygons (n = 938) in the Mediterranean region. The eight countries considered in this study are depicted in light gray (Algeria, Croatia, France, Greece, Italy, Libya, Morocco, Tunisia). Mediterranean countries in which the IWC is carried out but for which no site was selected are depicted in dark gray. The number and percentage of sites with polygons per country is provided in Appendix S1.

Appendix S4: Representation of protected sites and sites of international importance for waterbirds in our study sample compared to other samples.

|  | All sites  (n = 5891) | Coastal sites  (n = 1474) | Study sample  (n = 938) |
| --- | --- | --- | --- |
| Protected sites | 42.6% (n = 2507) | 50.4% (n = 743) | 59.9% (n = 562) |
| Sites of international importance for waterbirds | 4.5% (n = 268) | 6.4% (n = 94) | 6.5% (n = 61) |
| *C2* | 2.7% (n = 157) | 4.0% (n = 59) | 4.1% (n = 38) |
| *C5* | 1.6% (n = 94) | 2.2% (n = 33) | 2.6% (n = 24) |
| *C6* | 2.7% (n = 158) | 3.3% (n = 49) | 3.4% (n = 32) |

Appendix S5: Workflow used to model coastal flooding with ArcMap. Example with SSP5-8.5 (+77 cm).

**Step 1 – Reclassify the DEM:** 1 below the specified sea-level (here 77 cm), NA above


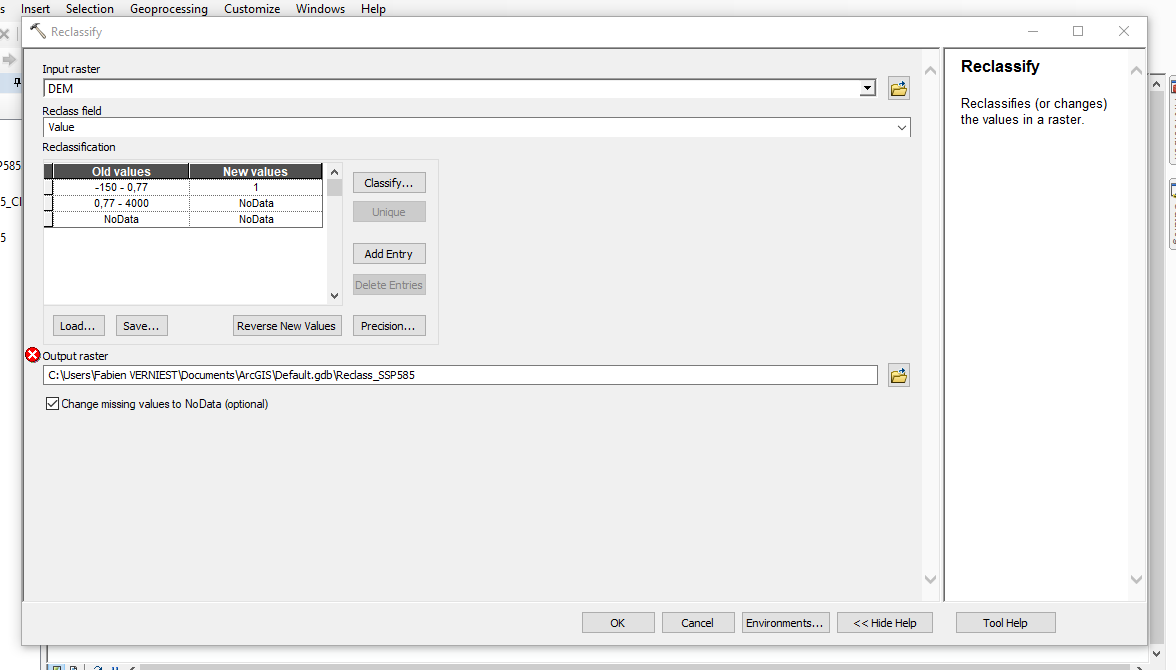


**
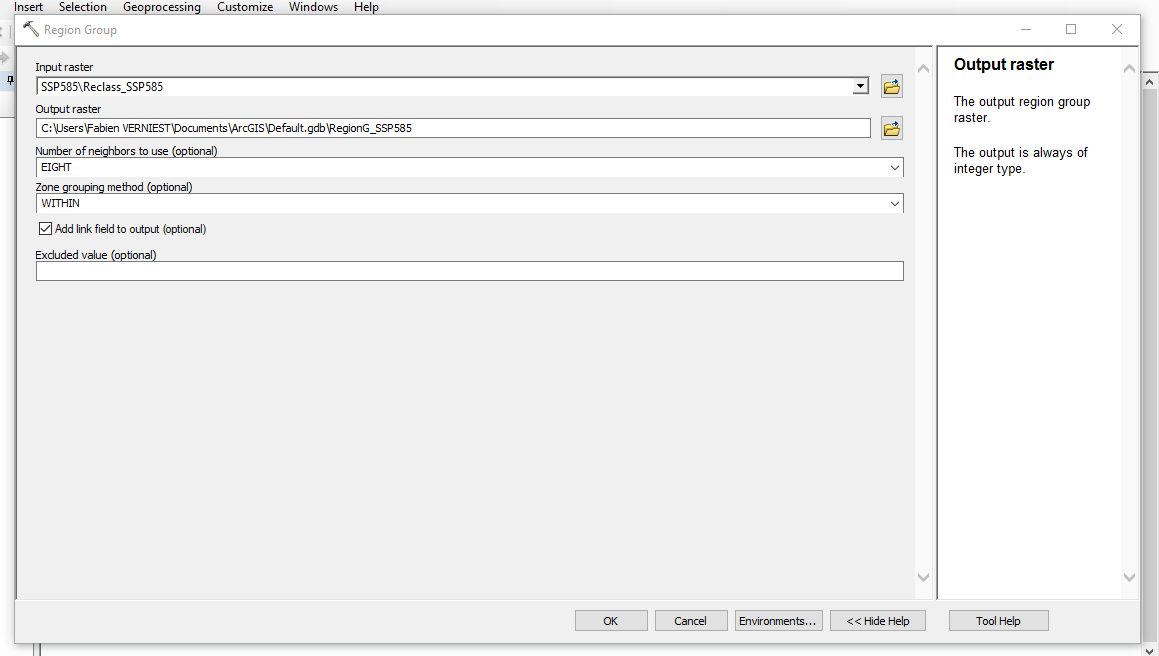
Step 2 – Region group:** Grouping of neighbouring pixels with a value of 1

**
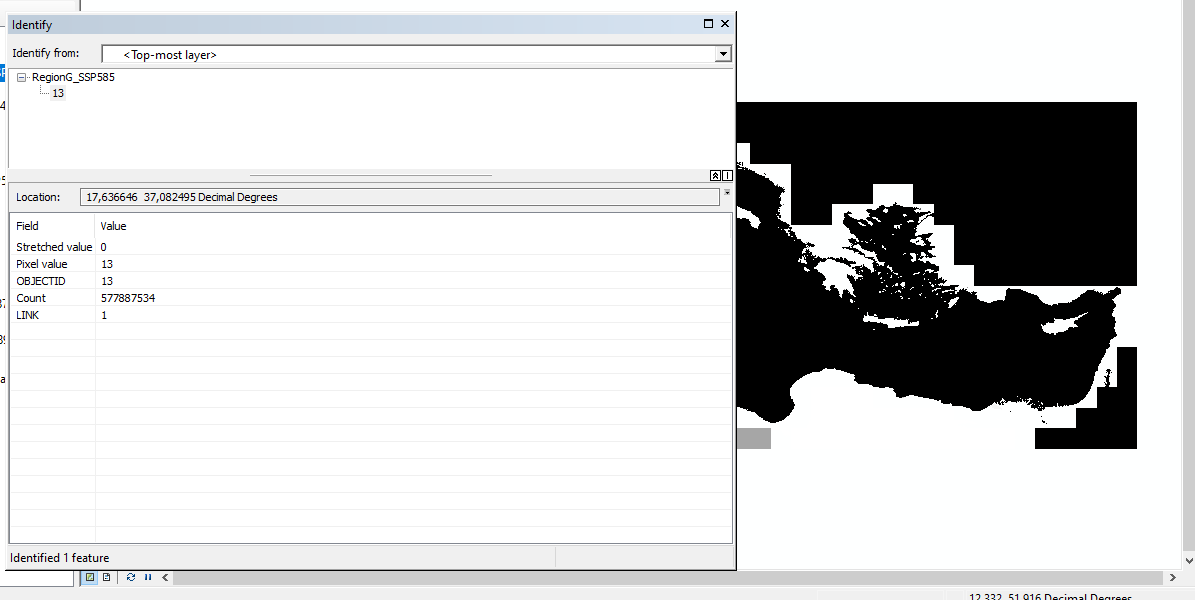
Step 3 – Identify the Mediterranean Sea group:** Value of the group of neighbouring pixels with a value of 1 corresponding to the Mediterranean Sea with +77 cm (here 13)

**
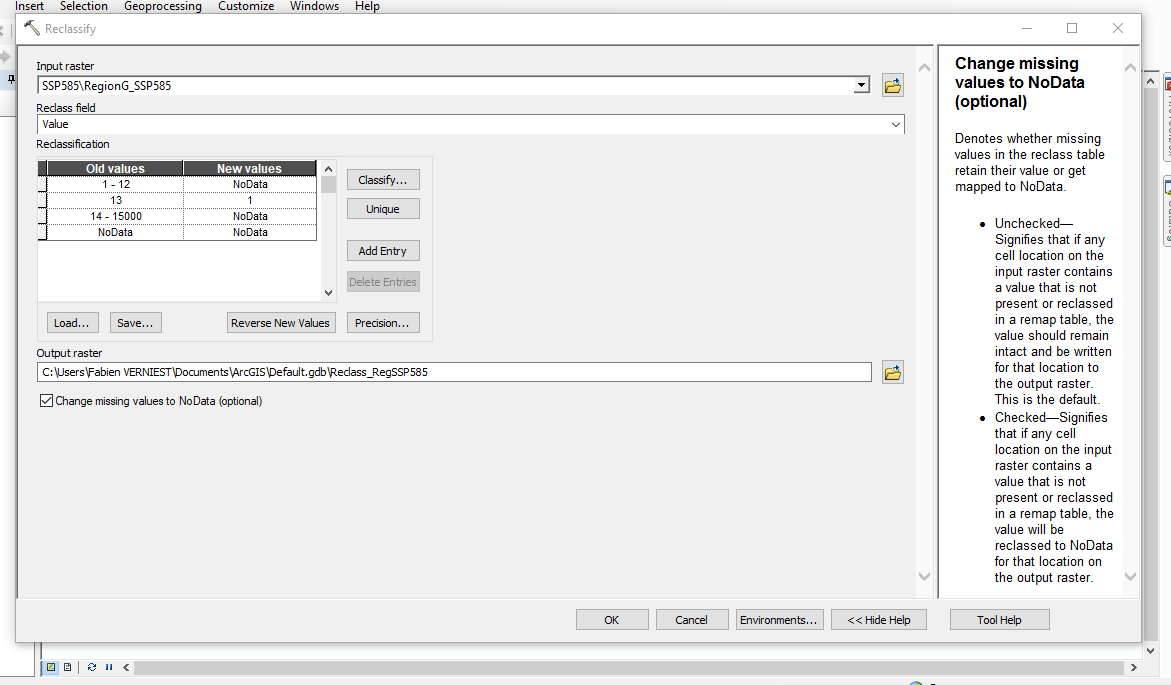
Step 4 – Reclassify the grouping:** 1 for the Mediterranean Sea, NA for the other groups

**
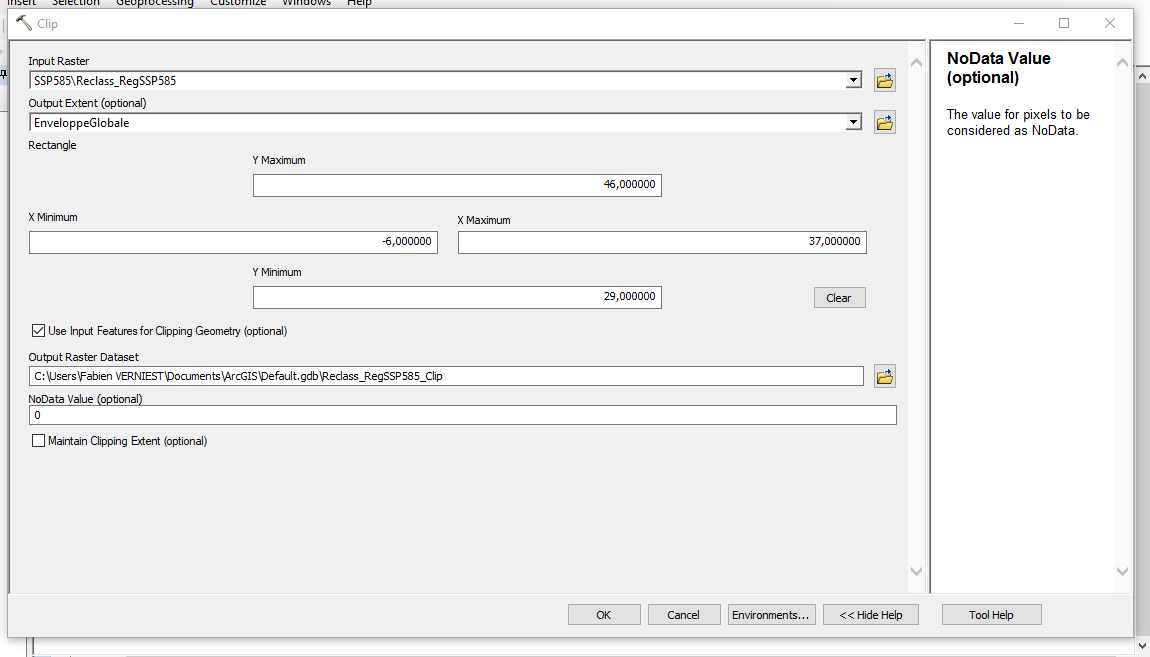
Step 5 – Clip the Raster using the study zone envelope**

**Step 6 – Export into Raster format**

**
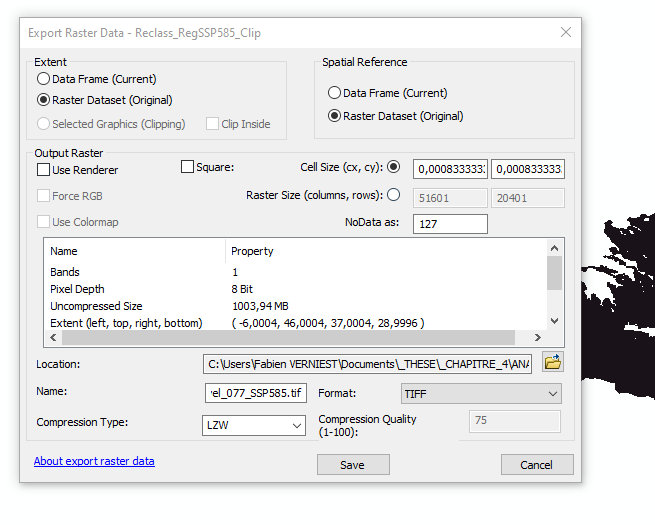
**


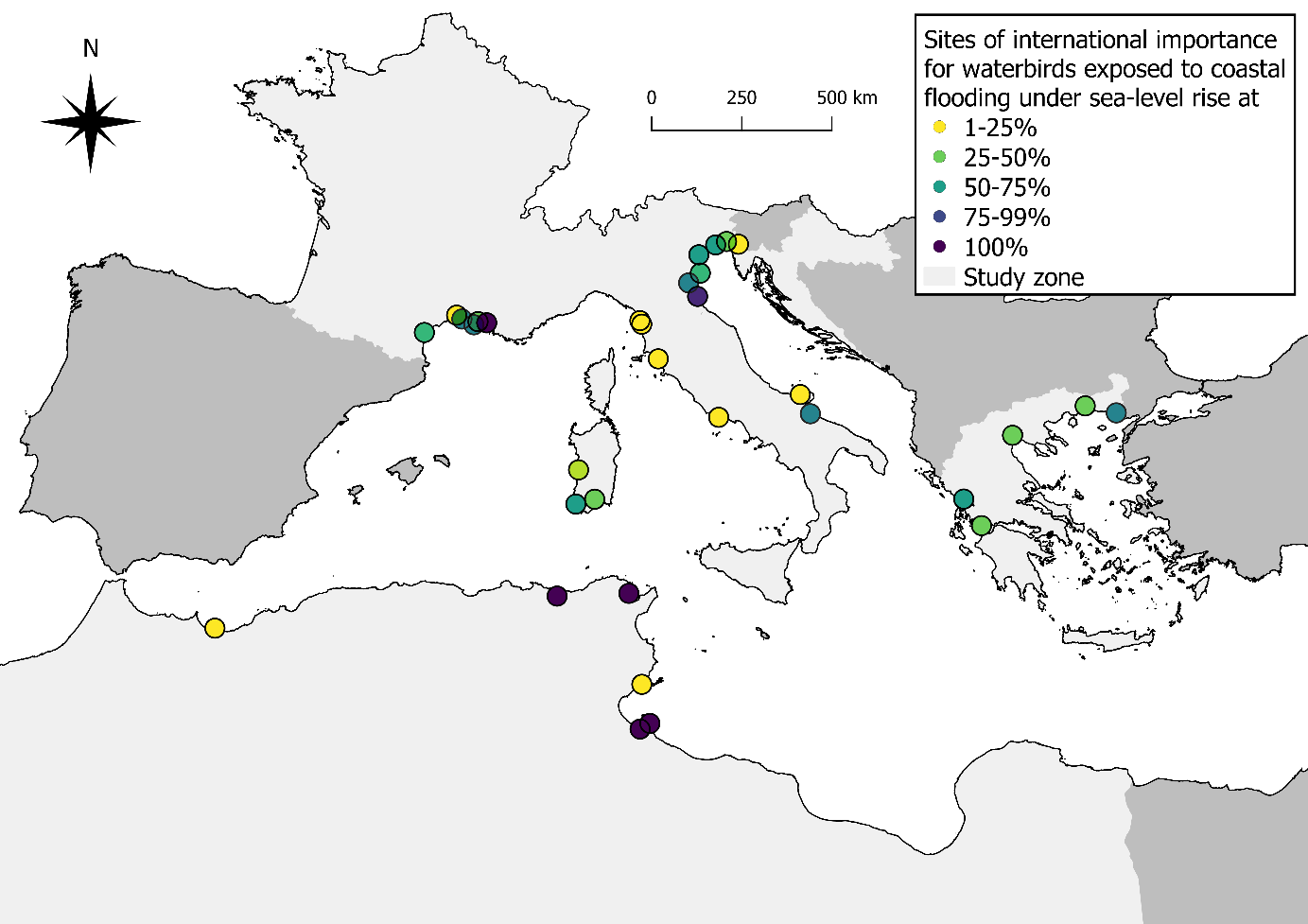


Appendix S6: Map of studied sites of international importance for waterbirds that are exposed to projected sea-level rise (Exp_SLR_ > 0%) in the Mediterranean by 2100 under SSP1-2.6. The eight countries considered in this study are depicted in light gray (Algeria, Croatia, France, Greece, Italy, Libya, Morocco, Tunisia). Mediterranean countries in which the International Waterbird Census (IWC) is carried out but for which no site was selected are depicted in dark gray.

Appendix S7: Effects of status of international importance for waterbirds on the exposure to coastal flooding under sea-level rise (Exp_SLR_) assessed for each scenario using beta regressions. Estimates are reported in bold when the 95% confidence interval does not include zero.

| **Scenario** | **Variable** | **Estimate** | **SE** | **2.5%** | **97.5%** |
| --- | --- | --- | --- | --- | --- |
| SSP1-2.6 (+44 cm) | Importance: Yes | **0.382** | 0.155 | 0.077 | 0.686 |
| SSP2-4.5 (+56 cm) | Importance: Yes | **0.392** | 0.161 | 0.077 | 0.706 |
| SSP3-7.0 (+68 cm) | Importance: Yes | **0.392** | 0.163 | 0.072 | 0.711 |
| SSP5-8.5 (+77 cm) | Importance: Yes | **0.398** | 0.165 | 0.074 | 0.722 |
| SSP5-8.5 LC (+88 cm) | Importance: Yes | **0.355** | 0.168 | 0.026 | 0.683 |
| SSP5-8.5 high (+102 cm) | Importance: Yes | 0.321 | 0.170 | -0.011 | 0.654 |
| SSP5-8.5 LC high (+161 cm) | Importance: Yes | 0.225 | 0.175 | -0.119 | 0.569 |


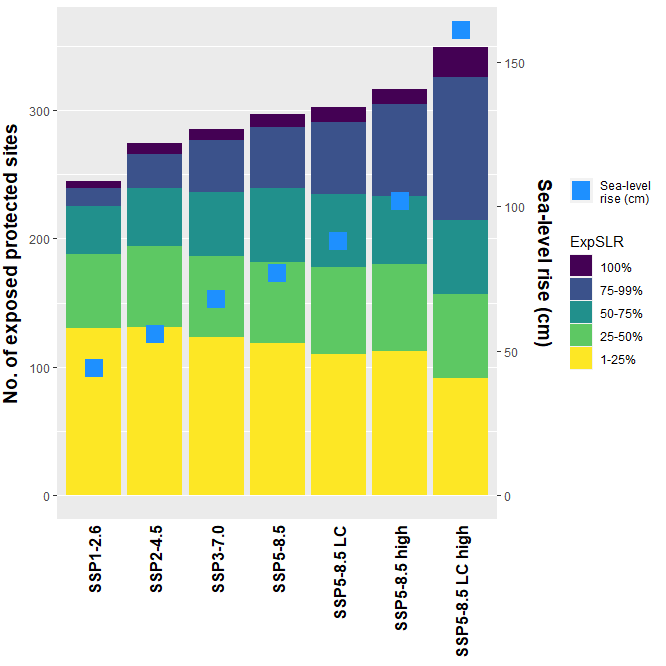


Appendix S8: Number of protected sites that are exposed to projected sea-level rise in the Mediterranean by 2100 (left axis) and projected sea-level rise (blue squares, right axis) under each scenario.

Appendix S9: Effects of protection status on the exposure to coastal flooding under sea-level rise (Exp_SLR_) assessed for each scenario using beta regressions. Estimates are reported in bold when the 95% confidence interval does not include zero.

| **Scenario** | **Variable** | **Estimate** | **SE** | **2.5%** | **97.5%** |
| --- | --- | --- | --- | --- | --- |
| SSP1-2.6 (+44 cm) | Protection: Yes | **0.215** | 0.076 | 0.067 | 0.363 |
| SSP2-4.5 (+56 cm) | Protection: Yes | **0.229** | 0.078 | 0.077 | 0.382 |
| SSP3-7.0 (+68 cm) | Protection: Yes | **0.246** | 0.079 | 0.091 | 0.400 |
| SSP5-8.5 (+77 cm) | Protection: Yes | **0.281** | 0.080 | 0.125 | 0.438 |
| SSP5-8.5 LC (+88 cm) | Protection: Yes | **0.251** | 0.081 | 0.092 | 0.410 |
| SSP5-8.5 high (+102 cm) | Protection: Yes | **0.247** | 0.082 | 0.085 | 0.408 |
| SSP5-8.5 LC high (+161 cm) | Protection: Yes | **0.270** | 0.086 | 0.101 | 0.439 |
